# Organizational Change and Workplace Incivility: Mediated by Stress, Moderated by Emotional Exhaustion

**DOI:** 10.3390/ijerph20032008

**Published:** 2023-01-21

**Authors:** Muhammad Ali Raza, Muhammad Imran, Joanna Rosak-Szyrocka, László Vasa, Noor Ul Hadi

**Affiliations:** 1 Department of Management Sciences, COMSATS University Islamabad, Islamabad 45550, Pakistan; 2 Faculty of Management, Czestochowa University of Technology, 42-200 Czestochowa, Poland; 3 Széchenyi István University, 9026 Győr, Hungary; 4 College of Business Administration, Prince Mohammad Bin Fahd University, Al-Khobar 34754, Saudi Arabia

**Keywords:** organizational change, workplace incivility, stress, emotional exhaustion

## Abstract

Modern organizations continuously undergo change processes. The focus of the organizations remains on the macro level, but the micro level (i.e., employee’s perspective) is neglected. Using the conservation of resource theory (COR), this study examines the association between organizational change and workplace incivility. This study also proposes mediating and moderating mechanisms of stress and emotional exhaustion. The data were collected from 262 respondents working in public sector organizations in Pakistan using a time-lagged technique. The results proved that change significantly impacts workplace incivility. Moreover, stress mediates their relationship and emotional exhaustion moderates it. Furthermore, emotional exhaustion also moderates the stress–incivility relationship. Public sector organizations must focus on well-planned, inclusive, and adequately managed change processes to achieve the desired outcome; otherwise, adverse behaviors, including incivility, manifest. To the best of the authors’ knowledge, the organizational change and incivility relationship has not been explored in the past. Additionally, their relationship with stress and emotional exhaustion also requires empirical investigation. This study also adds to the literature on the conservation of resource theory.

## 1. Introduction

Organizations nowadays are in a constant state of change. An ample amount of research has been performed in the past few decades to understand the dynamics of change management. Organizations face continuous pressure to change to keep up with the turbulence and change in the environment. Organizational change has been found to have a significant relationship with many elements of organizations, including organizational learning, organizational justice, change efficacy, communication, logistics and support systems, job commitment, job satisfaction, job demand, and social relations at the workplace [1,2,3,4,5,6,7,8,9]. However, some studies have also found that organizational change does not yield the anticipated results. Organizational change can sometimes fail to produce the desired results [10,11]. One of the major reasons for the failure of organizational change is focused on technical aspects such as information systems and organizational structure, while less to no importance is given to the human resource aspect [12,13,14].

In addition, the focus of research on organizational change has been on macro level phenomena while the micro level has received little attention. Organizational change research has predominantly focused on the improvements that change can bring to the organization, the change process required, and how to deal with the change process. One of the major aspects that is missing is the impact of change processes on employees. Oreg et al. [15] established that an organization cannot be evaluated without considering the individuals (employees) working in it. The success of the change process depends on how employees perceive the change and their reaction to it. The change process cannot be successful no matter how many resources are invested in it if the employees do not commit to it [16].

Employees sometimes do not understand how to react to the announcement of the implementation of a change process. They go through mixed emotions including uncertainty, anger, despair, stress, and fear. This range of emotions being experienced by employees can result in the depiction of different behavioral patterns. One such behavior is workplace incivility (WI). “Workplace incivility is low-intensity deviant behavior with an ambiguous intent to harm” [17]. Uncivil behaviors encompass a lack of the use of basic etiquettes of politeness such as saying “thank you” and “please”, whereby brusque and rude language is used instead, thereby showing a lack of respect for colleagues and leaders [18]. Such uncivil behaviors are minor and mundane in everyday operations but tend to have consequences that cannot be ignored.

Although workplace incivility is lower in impact than overt workplace deviance, including harassment, workplace violence, workplace bullying, and aggression [17,19], it still has a negative impact on the organization. It not only tempers the working environment but also has a negative impact on profitability. WI negatively impacts the reputation and corporate image of the company and hampers the operational efficiency of the company, thereby reducing its ability to earn profits. In total, 98% of employees have reported having experienced incivility at the workplace, with half of them facing incivility on a weekly basis [17]. WI may be amplified during the implementation of the change process as employees are uncertain about the impact and outcome of the change process. However, the argument made has scant research available and therefore requires empirical investigation.

Organizations nowadays continuously undergo change processes. Employees must adjust themselves to keep up with the everchanging dynamics, which can result in strain and stress not only for the organization but for employees as well. Stress is a well-known factor in organizations and can result in low motivation, low performance, high turnover, workplace deviance, uncivil behaviors, poor communication, and conflicts [20,21]. Chusmir and Franks [22] highlighted that all the above factors in one way or another are related to stress and can have a negative impact on an organization’s performance. The implementation of organizational change may result in employees going through stress, which ultimately triggers workplace incivility.

Employees going through organizational change are like a ship going through waters during a storm as the process is uncertain and there is always the danger of the unknown [23]. Organizational change demands high emotional and physical commitment from employees which can trigger burnout, which Maslach [24] states can manifest in the form of emotional exhaustion. During the change process, employees may experience stress and, when amplified by the emotional exhaustion factor, they may show incivility. This notion, however, requires empirical investigation. We posit that employees high in emotional exhaustion may behave differently than employees that are low in it.

The study contributes to the existing literature on organizational change management, stress, and emotional exhaustion in multiple ways. Firstly, to the best of our knowledge, the relationship between organizational change and workplace incivility is still unexplored, so it is pertinent that the said relationship be empirically examined. As the relationship is unexplored, the mediation between the two variables has also not been examined. The stress and workplace incivility relationship has been explored before [22,25,26,27,28,29,30,31], and the organizational change and stress relationship has also been evaluated [7,32,33,34,35].

However, all the studies mentioned have not examined the impact of organizational change on workplace incivility by taking stress as a mediator, and our study strives to achieve that. Secondly, emotional exhaustion has not been tested as a moderator between stress and workplace incivility. The aim of the current study is to examine EE impact as a moderator between stress and WI. Thirdly, as Raza et al. [36] mentioned in their study, the public sector is of pivotal importance for job creation in Pakistan, so the current study also strives to explore the said relationships in the public sector, which have not been explored before. Public sector organizations are different from private sectors especially in a country such as Pakistan which is characterized by a high power distance and collectivism [37]. Moreover, workplace deviance, incivility, and aggression cases have been reported in Pakistan [38]. Therefore, results based on a sample collected from the public sector would provide insight that is missing in the current literature. A time-lagged approach was employed for data collection as the change process is not a single event; rather, it is a series of instances, so a time-lagged approach is better suited for the current research. Lastly, predominantly, the research that exists has a Western perspective. The current study aims to evaluate the identified relationships from an Eastern perspective by taking a sample from Pakistan.

## 2. Literature Review

The current study is built on the premise of the conservation of resource theory [39]. The COR theory posits that individuals tend to acquire, maintain, or preserve objects, energies, personal characteristics, and conditions. Stress occurs in the workplace when employees are confronted with threats or the loss of identified resources [39]. As per the COR theory’s first principle, resource loss is disproportionately more salient than resource gain and employees are more sensitive to such stress-creating factors. Organizational change can lead to the redistribution, readjustment, and even reduction in resources and also a shift in the current state of operations.

### 2.1. Organizational Change

Organizational change can be defined as the deliberate effort of an organization to move from the current state to a desired state [40,41]. Organizations initiate change processes for adopting new strategies, changing or fine-tuning employment dynamics, and adjusting the structures used in the organization in order to keep up with their contemporaries [9]. The focus of companies on the continuous improvement and advent of concepts including total quality management and six sigma have also highlighted the importance of change management and its impact on organizational performance [2]. Kurt Lewin first coined the concept of change in 1948. Lewin, in 1948, established that human conflicts need to be resolved in order to enhance human conditions [40]. Lewin’s three-step change model is considered one of the fundamental models of change that emphasizes unfreezing from the old state, transitioning to a new desired state, and making it a permanent part of the organization. Modern-day change interventions focus on various aspects, including strategic, technostructural, and human process changes [42].

To obtain the desired outcome from change intervention, it is imperative that different aspects of intervention including the resources, time, and individuals involved in the change process are identified. Organizations need to develop an integrated approach to steer a constructive change and minimize the negative aspects while also addressing the consequences of the change process [11]. Organizations have reported having less than a 30% success rate of change processes [10,43,44], and further research has shown that the success rate is not getting any higher [45,46,47,48].

Because of the low success rate, employees going through the change process experienced several emotions, the majority of which were undesirable. Due to such negative emotions, employees depicted behavioral patterns that could hamper the change process. Skeptical of the planned change, employees may both actively and passively take steps to resist the change process. This can result in an unsuccessful change process, which ultimately leads to low morale and productivity [4,49], increased turnover, and high chances of organizational failure [49,50].

It is the responsibility of the organization to identify the environmental factors that are necessary for the change process to succeed. For the change process to be successful, a positive employee attitude is pivotal. Organizations need to create such an atmosphere where collaboration and trustful communication can take place, which may serve to achieve organizational change goals [7,9,51]. Management needs to ensure that employees can trust their leaders, which may reduce the feelings of uncertainty, fear, and anger. Employees who trust their leaders better align with the managerial values and are more receptive to organizational change [42].

Studies have shown that the chances of success of change processes are increased when employees feel supported during the change process [52,53,54,55]. Moreover, organizations that have a conducive environment for innovation and risk taking have better chances of successfully implementing planned changes [2,4,5]. Organizations need to create a supportive environment for change and reward improvements occurring in employees because of the change process. Creating such links may not only support the change process but may also diminish chances of adverse and deviant employee behavior because of the change process.

### 2.2. Instigated Workplace Incivility

Workplace incivility can be classified as “low intensity deviant behavior with an ambiguous intent to harm the organization” [17]. These behaviors are in violation of the norms of mutual respect at the workplace. Uncivil behaviors are basically discourteous and rude with a lack of regard for others [19]. There has been confusion between workplace incivility and other negative behaviors including aggression, antisocial behaviors, resources theft or misuse, and violence [17]. However, there is a fundamental difference in the aspects, including the targets, intention to harm, intensity, and nature of the violation [56]. Aggression with a clear intent to harm from the perpetrator can be differentiated from incivility with an unclear intent that can be attributed to other factors including personality, confusion, or a mere coincidence [17]. Any insensitive, disrespectful, or rude behavior at the workplace that violates social norms, though it may or may not be intentional, can be admitted as incivility [57,58].

Uncivil behaviors are subtle rather than overt, verbal rather than physical, passive rather than active, and indirect rather than direct. Given the low intensity, the perpetrator may deny any such intent and may cause harm accidentally rather than deliberately. Although the behavior is low in intensity, the impact can be severe as it may lead to severe aggression and continuing interpersonal conflicts [17]. Uncivil behaviors are considered one of the most harmful treatments that employees have to encounter at the workplace [59]. Porath et al. [60] and Schilpzand et al. [61] have empirically proven that incivility has a negative impact on employee performance by inhibiting work engagement, performance, and creativity at workplace.

Pearson and Porath [62] highlighted that contemporary organizations are characterized by continual change, and because of this, employees face certain pressures. Employees working in contemporary organizations are exposed to various organizational changes including restructuring, downsizing, rightsizing, continuous improvement, complex tasks, different working arrangements (compressed working hours, flexible timings, deadlines), and reformed psychological contracts (lack of job security and employability) [63]. Due to such continuous changes in the work environment, employees may experience higher levels of stress and anxiety, which ultimately results in rising levels of incivility. Raza et al. [36] also established that employees working in public sector enterprises in the tourism industry show higher levels of incivility because of everchanging policies resulting in a lack of organizational justice. Based on the literature, the first hypothesis of the study is:

**Hypothesis** **1** . *Organizational change leads to workplace incivility*.

### 2.3. Stress as Mediator

Stress has been a major topic for researchers and practitioners in the fields of health, medicine, psychology, and organizational behavior. Sackey and Sanda [64] and Wright [65] established that stress has a negative impact on employee job performance, mental health, and psychological wellbeing, which can lead to further complications. Stress not only influences health but also hampers the day-to-day operational efficiency of employees. Stress also increases ill health, the number of accidents at the workplace, and employee turnover intention, all of which in totality hampers the operations and ultimately results in lower efficiency and profitability [66]. Emotional exhaustion, cynicism, a lack of interest, and a lack of accomplishment are some of the outcomes for employees going through stress [67].

Elrod and Tippett [68] and Grant [69] established that change can be very stressful for employees going through it and can be classified as a stressor. Emotions and responses to change can be so intense that they have been compared with death and grief in the literature on organizational change [35]. Such reactions are normal as employees move from a state of known to unknown (Bovey & Hede, 2001. Consequent of the stress that employees face because of change, various behavioral patterns surface, one of which may be incivility [33,34]. There is a significant relationship between employees’ sense of control and stress. A greater sense of control results in a lower level of stress and vice versa [65]. Stress that manifests itself in the form of stressors was found to have a transitionary impact that occurs as a response to organizational change, which leads to adverse responses from employees. Stressors impede employees’ ability to function properly, which adversely impacts their ability to perform [33]. During the change process, employees feel a lack of control because of uncertainty; this causes stress, which can ultimately result in adverse behaviors including incivility. So, our next hypothesis is:

**Hypothesis** **2.** *Stress mediates the relationship between organizational change and workplace incivility*.

### 2.4. Emotional Exhaustion as Moderator

Emotional exhaustion can be classified as “the feeling of being emotionally extended and exhausted by one’s work”. Emotional exhaustion manifests in the form of being both psychologically and emotionally drained and physically fatigued [23]. Historically, work on emotional exhaustion stemmed from Maslach [24], who conceptualized burnout based on three components: emotional exhaustion, diminished personal accomplishments, and depersonalization. Depersonalization is best understood in customer-care-related jobs in which the workers depict callous insensitive behavior towards the client. Diminished personal accomplishments refer to the negative evaluation of oneself in which a worker feels as though they are ineffective and incompetent. Employees feel a lack of an ability to substantially contribute and thereby give themselves low ratings [23]. It is also pertinent to understand that emotional exhaustion is a component of burnout while stress is a standalone variable.

Stress and emotional exhaustion are inherently different concepts as stress is temporary in nature and manifests itself as a reaction to events and changes occurring at the workplace while, on the other hand, emotional exhaustion is relatively permanent in nature [70]. In addition, stress surfaces more as a reaction, which ultimately leads to responses such as deviance and incivility, while emotional exhaustion impacts the relationship as an independent factor [70].

**Figure 1:**
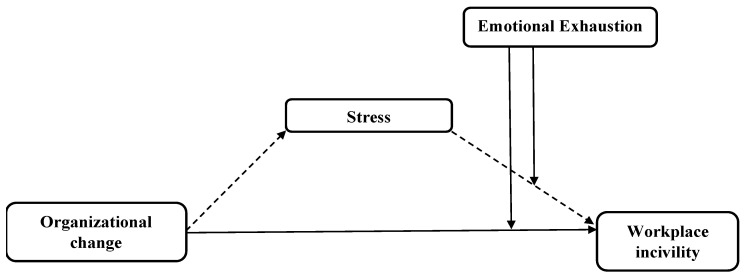
Conceptual framework.

Changes in organizational policies and structures including downsizing and mergers cause stress and burnout [7]. At the very core of stress and burnout is the premise of emotional exhaustion depicting continuously depleting emotional resources. It is highly chronic and causes adverse outcomes including incivility. When dealt with early in the burnout phase, emotional exhaustion can be effectively managed [71]. Emotional exhaustion is found to be more reactive, especially in the case of change in work settings. Change processes are mostly complex and sequential activities that require certain knowledge and skill sets. Moreover, even if the change process is implemented as planned, it still does not guarantee success; therefore, employees experience certain emotions that can hamper the change process itself or can also trigger or aid in triggering behavioral patterns that are adverse for the organization. Baba et al. [72] established that emotional exhaustion has a moderating effect and adversely impacts employees’ performance. Figure 1 is devised on the basis of the arguments made. Keeping the argument in mind, our next hypotheses are:

**Hypothesis** **3a** . *Emotional exhaustion moderates the relationship between organizational change and workplace incivility such that employees that are high in emotional exhaustion show higher levels of workplace incivility compared to employees that are low in emotional exhaustion*.

**Hypothesis** **3b.** *Emotional exhaustion moderates the relationship between stress and workplace incivility such that employees high in emotional exhaustion show a higher level of incivility compared to employees that are low in emotional exhaustion.*

## 3. Research Methodology

The current study aimed to evaluate the causal relationship between organizational change and workplace incivility with the mediation of stress and moderation of emotional exhaustion. Time-lagged data were collected to test the hypotheses. Data for the independent variable and mediator were collected at T1 while data for the moderator and dependent variable were collected at T2. The span of time between the two intervals was one month. Raza et al. [42] used time-lagged data with a gap of four weeks to examine the impact of organizational change on turnover intention in a similar sector, so our study followed a similar process. The time-lagged technique was also suitable for the current research as it helped us understand employee behavioral patterns in response to the implementation of change processes, which also occurred over a period rather than at a single point. Moreover, the baseline level of the dependent variable was controlled for in the current study as we did not attempt to examine responses from the same individuals at different time intervals.

### 3.1. Population and Sample

The population for the current study was a public sector of Pakistan. Two public sector universities and two public sector hospitals were selected for the purpose of data collection. The public sector plays a pivotal role in job creation, and it employs 7.5% of the total workforce in Pakistan as per the report published in The News (2020) [73]. Data for the current research were collected using purposive sampling. G*power was employed for the calculation of the sample size. A regression analysis was carried out to test mediation and moderation and a slope test was used to evaluate moderation at high and low levels. So, a regression analysis with a slope test was entered into the software. An effect size (medium, 0.05) with an α value of 0.05 at 0.95 power and the number of predictors was set to one. The sample size given by the software was 238.

The sample size depends on a number of factors including calculations and the judgement of the researchers, as identified by Saunders [74]. Keeping the above consideration in mind, a total of 350 questionnaires were floated for data collection. The floated questionnaires were self-administered, and the data were collected with the help of an electronic questionnaire that was emailed to the respondents. 

### 3.2. Instruments

Primary data were collected for the purpose of this research. To measure organizational change, Armenakis et al. [75] used a twenty-five-item scale, which included items such as “The change will benefit me”. Stress was measured using Parker and DeCotiis’s [76] scale containing thirteen items, which included items such as “I have felt fidgety or nervous as a result of my job”. Schaufeli [77] used a five-item scale to measure emotional exhaustion, which included items such as “I feel emotionally drained from my work”. Workplace incivility was measured using Cortina et al.’s [27] seven-item scale, with the leading phrase being “have you witnessed, experienced or shown following behaviors at workplace?”. The scale included items such as “Someone put someone else down or was condescending in some way”. All the responses were measured on a five-point Likert scale with “1” being coded as strongly agree and “5” as strongly disagree. The data collected were in a noncontrived environment.

### 3.3. Analysis and Techniques

AMOS 22 and the statistical package for social sciences (SPSS V 23) were used for data analysis. Structured equation modelling fundamentally encompasses the analysis of two models: the measurement model and structural model. The measurement model examines the relationship between latent variables and their respective items. For this, confirmatory factor analysis was carried out. A factor loadings, reliability, and validity analysis were conducted to check the fitness of the measurement model. The structural model, on the other hand, examines the relationship between the variables conceptualized in the framework [78]. For this, a correlation and regression analysis were conducted to test the direct, mediation, and moderation effect. Hayes’ [79] Process macros for SPSS was used to test the moderation and mediation effects.

A confirmatory factor analysis was conducted to examine the distinctiveness of the variables. For this factor loading, convergent and discriminant validity were checked to see the appropriateness of the measurement model. In addition, model fit indices including the goodness of fit index (GFI), Parsimony comparative fit index (PCFI), comparative fit index (CFI), root mean square of error approximation (RMSEA), and PCLOSE were examined to check the overall model fit.

## 4. Analysis and Results

A total of 350 questionnaires were floated at T1, out of which 311 were received back. At T2, 311 respondents were sent the next part of questionnaire, and out of these, 293 were received back, giving a response rate of 83.7%. From the received questionnaires, 31 were discarded due to incomplete responses. Consequently, 262 questionnaires were available for analysis. We obtained such a high response rate because of the purposive sampling employed for data collection. The respondents were informed beforehand about the details of the research, and the questionnaires were floated to the respondents that volunteered to take part in the data collection process. Moreover, the sample size was scientifically calculated using G*power, which also supports the generalizability of research. The demographics of the respondents are given below.

Table 1 shows the demographics of the respondents. Out of 262 respondents, 169 were male (64.5%) and 93 were female (35.5%). In total, 140 respondents were 25 or under (53.4%) while 99 were between 26 and 40 (37.8%). A total of 17 respondents were between 41–55 (6.5%) and 6 were above the age of 55 (2.3%). Out of the total respondents, 169 had less than 3 years of experience (64.5%) and 65 had 3 to 5 years of experience (24.8%). In total, 25 respondents had 6 to 10 years of experience (9.5%) while 3 had more than 10 years of experience (1.1%). Regarding education, 35 respondents had higher secondary school certification (HSSC) (13.4%), 212 had a bachelor’s degree (80.9%), 8 had a master’s degree (3.1%), and 7 had a degree higher than a master’s (2.7%).

### 4.1. Confirmatory Factor Analysis

Four measurement models were developed to test the discriminant validity of our constructs. For model 1, all the items of OC, OS, EE, and WI were loaded on a single factor. Model 2 consisted of two factors: one with all the items of OC and OS and two with items of EE and WI. For model 3, three factors were developed, with factor one having all the items of OC, factor two having the items of OS, and factor three having the items of EE and WI. For model 4, the items were loaded on their respective variables. Model 4 gave the best fit indices. Hu and Bentler (1999) [80] thresholds were used to assess the fitness of the model. Out of all the models, model 5 was best fitted with the data (χ^2^/df = 1.38, CFI = 0.97, RMSEA = 0.05, GFI = 0.861, PCFI = 0.833, PCLOSE = 0.188). The values for all fit indices are given in Table 2.

Factor loadings for all the latent constructs and their respective items were examined. As suggested by Sharma et al. [81], items with loadings 0.6 or greater were retained. To calculate the validities of all the constructs, the mean shared variance (MSV) and average variance extracted (AVE) were calculated. As per the Fornell and Larcker (1981) [82] criterion, the value of AVE for all constructs was above the lower threshold of 0.5 and the AVE values were also higher than the MSV values establishing discriminant validities among the constructs. In addition, the square root of the AVE of the constructs was higher than their correlations with other constructs, showing good convergent validity. The composite reliability (CR) for all the constructs was above the value of 0.7, showing good reliability. The common method variance was examined using the Harman factor test which showed that the variance explained by first factor was 37%, which was far below the cut-off value of 50% identified by Podsakoff et al. [83].

### 4.2. Correlations

Table 3 shows the mean, standard deviation, and correlations of the variables. The table shows that OC had a significant correlation with WI (r = −0.206, *p* < 0.05). OS had a significant relationship with WI (r = 0.536, *p* < 0.01). In addition, EE was also significantly correlated with WI (r = 0.171, *p* < 0.05). All the relationships identified were in the expected directions.

### 4.3. Hypothesis Testing

Hypotheses for moderation and mediation were tested using Hayes’ [79] SPSS Process macros. Testing of moderation with (Hayes, 2017) macros was suitable as it provides the results for moderation at three levels (low = −1SD, medium = mean, high = +1SD). To test the moderation, model 15 was used. Table 4 shows the results of the moderation analysis while Table 5 shows the results of the mediation analysis. The results showed that OC had a significant impact on WI (β = 0.65, *p* < 0.01), supporting hypothesis H1. The results also showed that OS had a significant impact on WI (β = 0.21, *p* < 0.01). Moreover, the interaction term generated from OC and EE had a significant impact on WI (β = 0.12, *p* < 0.01), supporting hypothesis 3a, stating that EE moderates the OC–WI relationship. Additionally, EE as a moderator had a significant impact on the OS–WI relationship (β = 0.15, *p* < 0.01), providing support for hypothesis H3b, which states that EE moderates the relationship between OS and WI.

**Figure 2:**
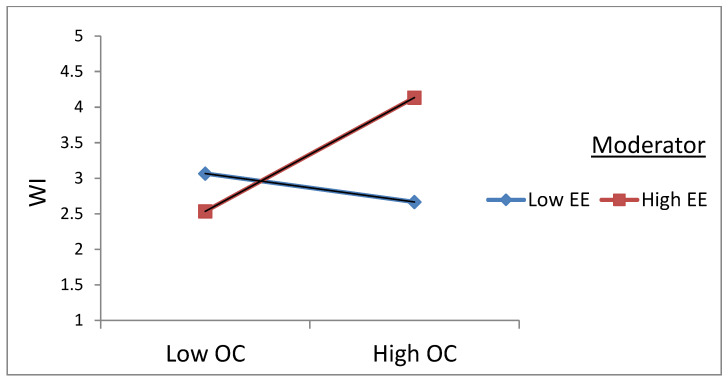
Interaction plot between organizational change and emotional exhaustion on workplace incivility.

In order to evaluate the nature of the moderations, interaction terms were plotted one standard deviation above and below the mean values A. Figure 2 shows the relationship between OC and WI at different levels of EE. The results showed that the OC–WI positive relationship was significant at a high level of EE (β = 0.32, *p* < 0.01) and the OC–WI negative relationship was also significant at low levels of emotional exhaustion (β = −0.07, *p* < 0.01), supporting the argument that employees low in EE do not show WI and rather support the change process. Hence, hypothesis 3a was supported.

**Figure 3:**
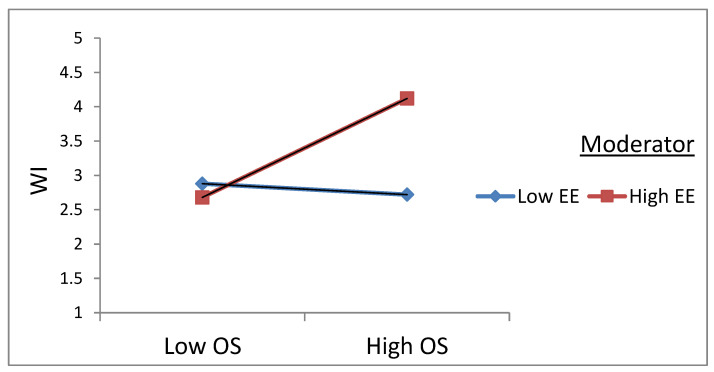
Interaction plot between Stress and emotional exhaustion on workplace incivility.

Figure 3 shows the relationship between OS and WI at different levels of EE. The results showed that the OS–WI relationship was positive and significant at a high level of EE (β = 0.41, *p* < 0.01) and that the OS–WI negative relationship was also significant at a low level of EE (β = −0.03, *p* < 0.01), hinting that employees low on EE support the change process. As a result, hypothesis 3b was supported, as EE moderated the OS–WI relationship. 

Table 5 shows the results of the mediation analysis. Model 4 of the Process macros given by Hayes [79] was used to test the mediation, and OC’s indirect impact on WI was evaluated through OS. The results showed that OC had a significant impact on WI (β = 0.25, *p* < 0.01) and that OC also significantly impacted OS (β = 0.10, *p* < 0.01). The results proved that the indirect effect of OC on WI via OS was significant (β = 0.05, CI [0.08, 0.20]) as there was no zero between the upper and lower limit; hence, H2, which stated that stress mediates the relationship between OC and WI, was supported.

## 5. Discussion

The aim of the current study was to examine the impact of organizational change on workplace incivility with the mediation of stress and moderation of emotional exhaustion. Based on the premise of the COR theory, our findings suggest that OC has a significant impact on WI. Employees experience several emotions during change processes, which induces them to show certain behaviors. Uncertainty, fear, and insecurity are some of the emotions that employee may experience during change as no matter how properly planned and managed it is, it can still be unknown territory for employees who, due to the experience of such adverse emotions, show negative behaviors. Employees that indulge in WI may not understand how to react to change and, with an ambiguous intent, show uncivil behavior. 

Although the current study is the first of its kind as the OC–WI relationship has not been explored previously, the findings of this study are somewhat in line with the findings of [17,53,60,62] as they mentioned that incivility is a reaction of employees to their work environment and the incidents taking place in it, and change may be one of such incident. This study also proved that due to organizational change, employees may experience stress, which supports previous research conducted by [7,33,35,42]. Stress is one of the key experiences that employees go through as a consequence of change processes, and it may trigger or aid in triggering certain behaviors in response to the change.

Our study also established that EE moderates the OC–WI and OS–WI relationship. When it comes to change and reactions to it, the employees’ emotional state plays a pivotal role. Due to cutthroat competition and focus on continuous improvement, organizations are in a constant state of change, which demands conviction and efforts from employees. This continuing spiral of change pushes employees into a chronic state of emotional and physical depletion which further escalates chances of employee incivility. Employees that experience emotional exhaustion exhibit workplace incivility, and on the other hand, employees that are not emotionally exhausted support the change process.

To sum up, our research aligns with the COR theory’s argument outlined by Hobfoll [39] that employees threatened by the fear of losing control over resources tend to conserve them. EE in such employees may alleviate their efforts to preserve the available resources.

### 5.1. Conclusions

The aim of the current study was to examine the impact of organizational change on workplace incivility with the mediation of stress and moderation of emotional exhaustion. The results proved that organizational change plays a pivotal role in workplace incivility. Moreover, organizational change stimulates stress, which leads to incivility, and the behaviors are further enhanced by emotional exhaustion, due to which employees lose interest in the organization. Organizational change coupled with emotional exhaustion also mentally and emotionally drains employees. If not dealt with, incivility can hamper the organization’s ability to reap the benefits of the change process, thereby making the complete process futile. Management must ensure that not only the change process is implemented successfully but that targets of the change process are communicated with employees to avoid incivility.

### 5.2. Theoretical Contribution

Our article makes theoretical contributions in at least in four ways: First, the relationship between organizational change and workplace incivility has not been studied before. Our study is an attempt to explore the relationship between organizational change and incivility and examine the relationship between the said variables. The second contribution of our research is incorporating stress as a mediator between OC and WI. Although stress has been studied prior in relation to OC [7,33,34,35,42], and also in relation to WI [28,29,84], its impact as a mediator between change and stress has not been examined. This current study is an attempt to contribute to the literature on stress by examining its mediating effect.

Third, we extended the application of the COR theory in a public sector organization context. Public sectors, especially in the context of a developing country such as Pakistan, are important as they help to uplift the economic condition by providing job opportunities. Conventional public sector organization employees resisted change and were against it in their efforts to conserve resources and due to the fear of a loss of power, authority, and incompetence. Such practices have made these organizations a load on exchequers’ pockets due to their outdated techniques, mechanisms, and operations. However, to keep up with contemporary needs, change has become inevitable for public sector organizations, and managing it is equally important. Employees experience various emotions during the change process, and if not properly managed, the entire change practice can go to waste due to adverse behaviors by employees.

Fourth, the study theoretically contributes to the literature available on emotional exhaustion. Employees, depending on their emotional and physical state, react to change. Drained by the continuous demand for improvement at the workplace, employees may not only lose their interest in the change process but also subconsciously or unconsciously show such behaviors that hamper the change. Our findings suggest that emotional exhaustion can act as a pivotal factor when it comes to the reception of change by their employees and their consequent reactions.

### 5.3. Practical Implications

The current study provides multiple suggestions for organizations currently going through change or ones that want to implement it in the near future. Employees are not comfortable with change processes and depict adverse behaviors as a reaction. Management must ensure that they properly communicate the need for change and that employees can be made a part of the planning process for change, whereby management takes the employees’ input and reduces the probable adversity to the announcement and implementation of change. Conviction for the change process can be obtained from employees by making them part of the change process as it would help them to feel ownership of the mechanism. Stress and emotional exhaustion may manifest in employees that are going through change. Such emotions need to be properly handled and dealt with to avoid incivility. Management can take suggestions from employees regarding their apprehensions for change and can also take necessary measures to remove any employee confusions or problems. An inclusive change process can be planned to avoid any roadblocks during the implementation stage.

Public sector organizations also need to nurture a culture that is open to change, and instead of creating hurdles in the change process, employees should commit to it. Innovation, creativity, and acceptance for change should be include in the culture of public sector organizations. Moreover, support and commitment for the change process should be incentivized.

### 5.4. Limitations and Future Research

There are certain limitations to our study that can be addressed by future researchers. The sample size was comparatively smaller as public sector organizations are not very open to data sharing and allow limited access. The data were collected in Pakistan only and future studies can collect data from multiple countries to conduct a comparative analysis on employees’ reactions to change. This study was based on cross-sectional data, and future research can use longitudinal data for better results. Emotional exhaustion was taken as a moderator in current study; however, for future studies, it can be examined as a serial mediator following stress, as the current literature provides merit for the examination of the identified relationship.

## Figures and Tables

**Table 1:** Demographic.

	Demographics	Frequency	Percentage
Gender	Male	169	64.5
	Female	93	35.5
Age	25 or under	140	53.4
	26–40	99	37.8
	41–55	17	6.5
	>55	6	2.3
Tenure	<3	169	64.5
	3–5	65	24.8
	6–10	25	9.5
	>10	3	1.1
Education	HSSC	35	13.4
	Bachelor’s	212	80.9
	Master’s	8	3.1
	>Master’s/Others	7	2.7

**Table 2:** CFA Summary.

Model	χ^2^ (df), *p*	CFI	RMSEA	GFI	PCFI	PCLOSE
Model 1	7451.321 (327), *p* < 0.01	0.37	0.22	0.479	0.511	0.000
Model 2	4490.584 (319), *p* < 0.01	0.61	0.17	0.716	0.631	0.021
Model 3	3011.71 (301), *p* < 0.01	0.70	0.11	0.781	0.723	0.321
Model 4	374.69 (270) *p* > 0.01	0.97	0.05	0.861	0.833	0.188

**Table 3:** Correlations.

	Mean	SD	Age	Gender	Education	Experience	OC	OS	EE	WI
Age ^a^	1.54	0.69	1							
Gender ^b^	1.35	0.47	0.073	1						
Education ^c^	2.92	0.53	0.169 *	0.135	1					
Experience ^d^	1.36	0.69	0.158	0.084	0.274 **	1				
OC	3.92	0.56	−0.027	0.160 *	0.245 **	0.132	1			
OS	3.50	0.77	−0.277 **	−0.014	0.085	−0.147	0.098	1		
EE	4.09	0.65	−0.120	0.061	0.247 **	0.110	0.576 **	0.187 *	1	
WI	3.49	0.80	−0.143	0.068	−0.034	−0.074	−0.206 *	0.536 **	0.171 *	1

* *p* < 0.05, ** *p* < 0.01, OC = organizational change, OS = stress, EE = emotional exhaustion, WI = workplace incivility. ^a^ = Age was coded as 1 = 25 or under, 2 = 26 to 40, 3 = 41 to 55, 4 = above 55. ^b^ = Gender was coded as 1 = male, 2 = female. ^c^ = Education was coded as 1 = matriculation, 2 = higher secondary school, 3 = bachelor’s, 4 = master’s, 5 = higher, 6 = others. ^d^ = Experience was coded as 1 = less than 3 years, 2 = 3 to 5 years, 3 = 6 to 10 years, 4 = more than 10 years.

**Table 4:** Moderation analysis.

	Β	SE	t Statistic	*p*	LL	UL
Independent Variable = OC						
Constant	3.1	0.22	5.51	0.01	0.80	2.10
OC → WI	0.65 **	0.58	4.35	0.01	−0.13	−0.29
EE → WI	0.48 *	0.53	4.41	0.05	0.15	0.49
OC × EE → WI	0.12 **	0.17	2.87	0.01	0.21	0.75
Age	0.05	0.08	1.45	0.08	−0.23	0.72
Education	−0.18	0.11	0.98	0.23	−0.15	0.18
Gender	0.09	0.11	0.70	0.04	−0.25	0.49
Experience	−0.02	0.08	0.72	0.21	−0.03	0.18
Conditional effects of moderator at M ± 1 SD (slope test)	Effect	SE	LL95% CI	UL95% CI		
EE Low −1 SD (3.44)	0.324	0.153	0.02	0.62		
EE Medium (4.09)	0.243	0.120	0.05	0.48		
EE High +1 SD (4.74)	0.162	0.146	0.12	0.45		
Independent Variable = OS	β	SE	t Statistic	*p*	LL	UL
Constant	3.1	0.22	5.51	0.01	0.80	2.10
OS → WI	0.21 *	0.49	3.41	0.01	0.27	0.98
EE → WI	0.48 *	0.53	4.41	0.05	0.15	0.49
OS × EE → WI	0.15 **	0.11	2.98	0.01	0.06	0.37
Conditional effects of moderator at M ± 1 SD (slope test)	Effect	SE	LL95%CI	UL95%CI		
EE Low −1 SD (3.44)	0.070	0.092	−0.10	0.18		
EE Medium (4.09)	0.059	0.074	−0.08	0.11		
EE +1 SD (4.74)	0.049	0.065	−0.06	0.10		

N = 262, LL = lower limit, UL = upper limit, CI = confidence interval, SD = standard deviation, M = mean, SE = standard error. * *p* < 0.05, ** *p* < 0.01. OC = organizational change, WI = workplace incivility, OS = stress, EE = emotional exhaustion.

**Table 5:** Bootstrapping results for direct and indirect effects.

Direct Effects		Coefficients	SE	t
OC → WI		0.25 *	0.10	2.47
OC → OS		0.10 **	0.11	3.47
OS → WI		0.55 *	0.07	7.48
95% bias corrected confidence interval method				
Indirect effect	Effect	SE	LL	UL
OC → OS → WI	0.05	0.07	0.08	0.20

SE = standard error. *p < 0.05, ** *p* < 0.01. OC = organizational change, OS = stress, WI = workplace incivility.

## Data Availability

Not applicable.
